# Insulating materials based on silica aerogel composites: synthesis, properties and application

**DOI:** 10.1039/d4ra04976d

**Published:** 2024-10-29

**Authors:** K. I. Goryunova, Y. N. Gahramanli

**Affiliations:** a Azerbaijan State Oil and Industry University, Department of Chemistry and Technology of Inorganic Substances Baku City Azerbaijan kristina.qoryunova.i@asoiu.edu.az

## Abstract

The rapidly increasing growth in the world's population has created enormous environmental issues, such as the greenhouse effect, global warming, ozone layer depletion, acid rain, and excessive energy usage. The construction sector, in particular, accounts for a significant share of worldwide energy consumption, making it a major contributor to these challenges. Effective building insulation is crucial for decreasing energy demand, minimising heat loss, and lowering environmental impact. Aerogels have acquired popularity as insulation materials due to their outstanding qualities such as great thermal insulation, flame retardancy, lightweight construction, and environmental friendliness. Silica aerogels, in particular, are emerging as game changers in the construction insulation sector, accounting for a sizable proportion due to their low thermal conductivity and simplicity of manufacture. This paper gives a thorough overview of silica aerogel production processes, the key qualities necessary for aerogel-based composites, and the most recent advances in their use in building insulation. It also examines how to overcome inherent obstacles such as mechanical fragility and high manufacturing costs, providing technologies that improve mechanical strength and long-term durability. By incorporating silica aerogels into construction materials, the industry may significantly reduce energy consumption while also contributing to the creation of sustainable, energy-efficient buildings.

## Abstract 2

This paper focuses on the most recent advances in silica aerogel-based composite research, and indicates novel applications as insulation materials.

## Graphical abstract

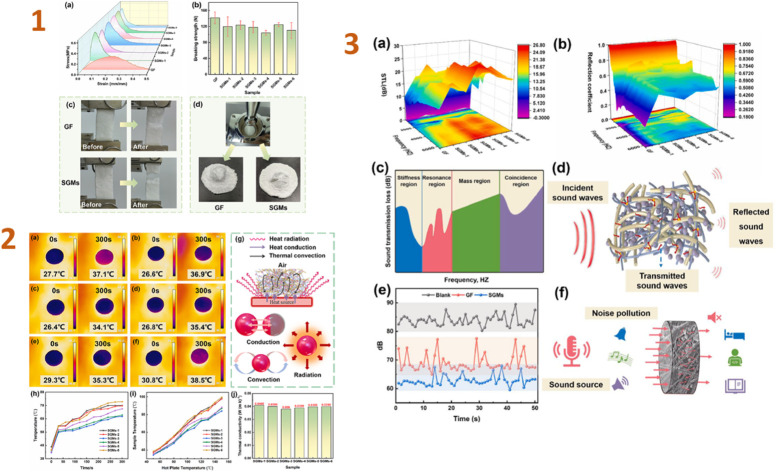

## 1. Introduction

The expansion of the population causes several ecological problems including the greenhouse effect, global warming, stratospheric ozone depletion, acid precipitation, and excessive energy usage.^1^ Furthermore, the majority of environmental issues are due to excessive energy use and increased CO_2_ concentrations.^2–4^ Global demand for energy is rising, with a projected 53% rise by 2030.^5^ Energy is mainly separated into two subgroups: renewable and non-renewable energy sources, with coal, oil, and natural gas falling into the second category. With rising human needs, the stocks of these non-renewable resources have dramatically increased greenhouse gas emissions over the last decades.

The construction and building industry has the highest consumption of energy and losses.^6–9^ Given that crude oil and natural gas resources are needed to offer thermal comfort, it is obvious that they should be used efficiently. In this context, building insulation is critical for minimizing energy use and expenditures. The insulation capabilities of buildings are determined by the qualities of the materials used for the structure's outside and interior faces, or building and structural parts. The thermal insulation capabilities of these construction materials are defined by their ability to prevent heat generated within the structure from passing through the thermal envelope.^10^ The implementation of methods of isolation can significantly reduce overall energy loss and CO_2_ emissions, depending on the kind of insulation and application depth.^11–14^

Facilities consume the majority of their energy through heating, ventilation, and air conditioning.^15^ As a result, in order to guarantee thermal stability, the construction sector consumes a lot of energy, which may be lowered with proper insulation techniques. Although the initial cost may seem significant, well-insulated buildings can pay for themselves several times over their lifetimes. Along with insulation, energy consumption, reliance on fossil fuels, and greenhouse gas emissions caused by energy usage will simultaneously decrease.

Heat insulation materials can be natural (*e.g.*, plant or animal wool, cellulose), mineral (*e.g.*, pumice, tuff, scoria), or synthetic/semi-artificial (*e.g.*, exfoliated vermiculite, expanded glass, expanded perlite, mineral wool).^16–21^ However, several of these traditional thermal insulating materials, such as sheep and mineral wool, polystyrene, and cork, have inherent disadvantages. Although many insulation materials have been studied in the past and their results described, products made from these materials typically need to be applied in thick layers to achieve adequate insulation values. As a result, these materials might reduce living space and cause aesthetic issues. Furthermore, some of them have poor fire resistance and durability issues.^22^ As a result, the need for new thermal insulation materials that can be placed in thinner layers has increased in recent years.

For several decades, building insulation has accounted for more than two-thirds of the total thermal insulation industry market. Aerogel materials are environmentally friendly, easy to prepare, flame retardant, and exhibit excellent thermal insulation efficiency.

Aerogels are solid, mesoporous materials with high porosity and low density. These materials exhibit a variety of distinguishing features, including transparency, a large specific surface area, low heat and acoustic conduction, mechanical endurance, and high sorption activity.^23^ The average thermal conductivity index of silica aerogels was 0.01 W m^−1^ K^−1^,^24^ making them perfect thermal insulators. As a result, scientists all around the world began to research aerogels in combination with other materials as a composite insulating material.

Researchers have undertaken extensive study on the silica aerogel heat transfer model in order to achieve intelligent design of silica aerogel composites. Previous studies attempted to develop models of multiple heat transfer mechanisms (gas, solid, and radiation heat transfer) in silica aerogel materials. Fricke first developed an equation model for silica aerogel thermal conductivity, established a mathematical relationship between silica aerogel density and heat transfer of gas, solid, or radiation,^25^ and considered silica aerogel's general thermal conductivity as an average of three-phase thermal conductivity. Lee *et al.*^26^ employed this foundation to provide a technique for calculating the gas phase thermal conductivity of silica aerogels. Zeng *et al.*^27^ estimated the average free route of gas molecules in aerogel materials. The link between the thermal conductivity of aerogel materials and gas viscosity may be deduced from molecular dynamics theory using the mean free path principle. This approach for estimating the total thermal conductivity of silica aerogels has a straightforward formula, but it has the disadvantage of include several empirical constants that have no special physical relevance.

Zeng *et al.*^28^ suggested three types of cubic arrays, namely, orthogonal square rods, orthogonal cylindrical rods, and orthogonal spherical arrays, to characterise the morphology of silica aerogels and determined a thermal conductivity model of silica aerogels using the corresponding circuit method, which was a significant advance for research on the thermal conductivity of silica aerogels. Zhao *et al.*^29^ created an improved analytical model for the overall thermal conductivity of silica aerogels and presented a three-dimensional cluster aggregation structure with random diffusion to compute the thermal conductivity of silica aerogels at various temperatures. This framework is a pure forecasting tool that can only analyse the thermal conductivity and high-temperature properties of silica aerogels by using microstructure measurements from tests and analysis as input parameters. Yu *et al.*^30^ used the similar circuit method to develop a semiempirical model for calculating the effective thermal conductivity of composite aerogel materials. This model takes into account the nanoscale heat transfer effect as well as the microscale influence of additives such as emulsifiers and reinforcement fibres, allowing for a more accurate prediction of effective thermal conductivity. Feng *et al.*^31^ developed a model to characterise the scale heat transmission process of fumed silica-based thermal insulation composites, and they investigated thermal conductivity, solid thermal conductivity, and radiation heat transfer.

This study describes common types of silica aerogel-based composites used nowadays, and overviewing both the advantages and disadvantages of them as the construction and building materials.

## 2. Synthesis

The production of silica aerogels has drawn a lot of interest and is extensively reported.^23,32–36^ Some studies investigated the application of various precursors, while others concentrated on modifying parameters associated with synthesis.^37–41^ Therefore, just a basic summary is provided here.

The procedures of an aerogel manufacturing may be divided into four primary steps:

(a) Formation of the sol.

(b) Gelation.

(c) Aging.

(d) Drying.

### 2.1. Formation of the sol and gelation processes

The silica sol is produced using the technique known as sol–gel. The sol is a colloidal mixture of silica particles that is formed by adding a base or acid catalyst, which causes pH fluctuations and extends the gelation time. The choice of silica precursor is critical in the sol–gel process. Precursors should be soluble and contribute to the gel forming process. Silicon alkoxides, such as tetramethoxysilane (Si(OCH_3_)_4_–TMOS) and tetraethoxysilane (Si(OC_2_H_5_)_4_–TEOS), are among the most popular precursors for silica aerogels.^32^

The low-temperature sol–gel procedure is the most commonly employed method for producing silica gels. In this process, hydrolysis and condensation reactions occur at ambient temperature, resulting in the production of a nanostructured silica network. Colloidal particles are formed as a result of a promoted chemical reaction between homogeneous molecular precursors that have been dissolved. In the sol stage, hydrolysis and condensation reactions occur in relation to the pH and temperature.

The solution pH influences the hydrolysis and condensation processes,^42^ resulting in significantly diverse gel topologies.^43^ When acid catalysts are utilised, hydrolysis proceeds quicker than condensation, resulting in a less branched silica network^34,43,44^ that is easily redissolved in aqueous solutions.^45^ In base-catalysed reactions, the converse is true, with the condensation step taking precedence over the hydrolysis stage, resulting in highly condensed networks with less remaining alkoxide and silanol groups as compared to acid-catalysed reactions.^34,44^ When the sol reaches the gel point, it is commonly thought that the hydrolysis and condensation processes have virtually completed.^33^

At the gelation stage, initial nanoparticles bind together to form and subsequently aggregate into clusters of a continuous network resembling a pearl necklace.^46^ The gels are named after the liquid solution that is enclosed within the silica pores. The gels are classified based on the pore liquid, such as alcogel (alcohol), hydrogel (water), and organogel (organic solvent).^47,48^

During the gelation procedure, a rise in the amount of –OH ions cause dehydration and condensation inside siloxane monomers. Gelation time is reduced by utilising greater concentration base catalysts, which supply more –OH ions and hence speed up the rate of condensation. At the gel point, the sol–gel transition is complete, indicating that the hydrolysis and condensation processes have ended. The aforementioned shift from liquid solution to solid phase is known as the sol–gel transformation. During this occurrence, primary particles are generated, which subsequently aggregate into secondary particles (clusters) before interconnecting in the pearl necklace morphology.^49,50^

### 2.2. Aging of the gel

The hydrolysis and condensation processes continue after the gel point has been reached, although at a considerably slower pace due to diffusional restrictions and a reduction in reactive species. This process is known as ageing, and the reactions cause the silica network to strengthen and stiffen, which has a substantial influence on the aerogel microstructure.^43,45^ The ageing process may be regulated or accelerated by adjusting several parameters such as solution pH, precursor concentration, and water content of the covering solution.^43,51,52^

The gel's network is strengthened by two primary mechanisms: the first is neck development, which results from the reprecipitation of dissolved silica from the secondary surface of the particle onto the neck region; the second is the Ostwald ripening mechanism, which involves the disintegration of smaller silica particles and then their reprecipitation onto larger ones.^33,34,53,54^

To improve the degree of cross-linking, the silica gel is frequently immersed in a solution containing silane precursors such as TMOS or TEOS during the ageing process.^43,45,53^ This method is comparable to surface modification and aids in the prevention of silica material fracture during drying at ambient pressure or at supercritical circumstances.^45,55,56^

### 2.3. Washing of the gel

Afterwards, a washing phase is normally done to remove the catalyst, debris, unreacted precursors, and any additives utilised, such as surfactants. The presence of these contaminants may cause partial collapse of the porous network after drying, increasing the gel's bulk density. This occurs because some of these chemicals, such as water, can interact with silanol groups on the silica surface, boosting capillary forces when drying. Washing is often accomplished by soaking the gel in pure alcohol in numerous phases and exchanging the liquid in the pores *via* a diffusion process.^51^

### 2.4. Drying of the gels

The final and most significant stage in the manufacturing of aerogels is drying the gel. The major goal is to preserve the gel's original pore structure after drying, which may be accomplished by minimising damage to the gel network induced by capillary forces during this process, hence improving the aerogel's characteristics.^57^ Three alternative drying processes are often used:

(1) Atmospheric pressure drying (APD).

(2) Supercritical drying (SCD).

(3) Freeze drying (FD).

APD technique is known as the simplest and cheap technique. However, this method has several drawbacks. When employing APD it is necessary to reduce capillary tension and pressure gradients, and in this case, solvent-exchange techniques are used, as well as gel surface modification (silylation). In the latter, the silylated surfaces resist each other, and no condensation processes occur after drying. Thus, the gel does not shrink irreversibly during this step and returns to its former porous condition after drying.^57,58^ This phenomenon is known as the spring-back effect.^51^ Because APD is performed at ambient pressure and moderate temperatures, it is less expensive and more versatile in terms of sample size than SCD and FD.

SCD was the first approach implemented to produce aerogels,^30^ and it is still the favoured method because supercritical fluids minimise surface tension effects during the drying process,^58^ considerably lowering shrinkage and collapse of the pore structure. There are two primary types of SCD:^57^ high temperature supercritical drying (HTSCD) and low temperature supercritical drying. The fundamental difference between the two procedures is the kind of solvent employed throughout the process; for example, the HTSCD method employs solvents with a high supercritical point, whilst the LTSCD employs the reverse.

In the FD process, the solvent in the pores is frozen before being sublimated under vacuum. The nanostructured matrix may break due to the development of massive crystals within the pores. This issue is exacerbated when water is used as a solvent since it expands when frozen, causing significant damage to the pore structure.^59^ This approach yields powder-like silica compounds with macropores.^34^

The FD technique is preferred for producing aerogel composites because it produces silica aerogels in powder form, which may be utilised as a filler, whereas the SCD approach is employed to produce monolithic silica aerogel composites.

## 3. Required properties of silica aerogel composites as insulation materials

The manufacturing of construction insulation materials includes an exact list of critical qualities, as well as the operating conditions necessary to reduce heat transmission, consumption of energy, and temperature control stabilisation. The most important property is the coefficient of thermal conductivity; other qualities include heat capacity, mechanical strength, density, porosity, dielectric strength, and so on.^60^

In this chapter we have reviewed only 3 mains but the most crucial properties required for production of composite materials.

### 3.1. Thermal conductivity

Thermal conductivity refers to a material's capacity to conduct heat. Heat is transported from one particle to another *via* molecular vibration, whereas energy is transferred by free electrons.^61^ Thermal conductivity of a material is determined by its lattice organisation, pore connectivity, and granule size.^62–64^

Thermal energy is transported *via* silica aerogels using three mechanisms: solid conduction, gaseous conduction, and radiative transmission.^34,65^ The effective thermal conductivity is computed by adding the thermal conductivities of each method of heat transmission and the heat transfer between gas and solids.

The intrinsic solid thermal conductivity of aerogels is determined by their network structure, connectivity, and chemical composition.^65^ This heat conduction occurs *via* phonon diffusion through the aerogel backbone, with a mean free path of 1 nm; hence, it is an isolated transport phenomena.^66,67^

Thermal energy can also be transported through the aerogel *via* the gas phase. The Knudsen effect is principally responsible for aerogels' low gaseous thermal conductivity.^65,68–70^

Theories for radiative heat transport are based on the Rosseland diffusion hypothesis.^71^ In addition, irregular additions can have an impact on the radiative heat transfer of silica aerogel composites. Mie's hypothesis^72^ was presented to mimic the effect of spherical additions. Lee and Cunnington's equation^73^ are shown for fibrous additives.

Different methodologies can be used to determine thermal conductivity, which is generally divided into two classes: (i) steady-state methods, which measure thermal properties by establishing a temperature difference that does not change over time, and (ii) transient-state methods, which typically measure the sample's time-dependent energy dissipation process.^74^ The most generally used techniques for determining the thermal conductivity of aerogel samples are the Guarded Hot Plate (GHP)^75–78^ in the steady-state case and the Transient Plane Source (TPS) approach^79–81^ for transient procedures.

Various standards, including ASTM C177,^82^ European Standard EN 12667,^83^ and International Standard ISO 8302,^84^ outline the apparatus and testing process for the GHP technique. Even though this technology is widely used, it has significant limitations, such as the need for relatively large testing samples and typically long wait times.^74^ The TPS approach, particularly the “Hot Disc” variations, has been used to quickly characterise thermal characteristics.^74,85,86^ The ASTM D7984 (ref. 87) and ISO 22007-2 (ref. 88) provide the instruments and processes for this technique. The TPS approach can measure thermal conductivities from 0.005 to 500 mW m^−1^ K^−1^ across a wide temperature range.^74^ However, the two sample pieces must be comparable and have one completely flat side,^89^ which might be difficult for aerogel samples.

### 3.2. Mechanical strength

Mechanical strength, or mechanical stability, is another key attribute of construction materials. Depending on compressive strength, composite materials may be categorised into three basic classes: (i) building material – can withstand pressures more than 10 MPa; (ii) construction insulation material – can withstand pressures up to 3 MPa; and (iii) insulation material – can withstand pressures between 0.5 and 1 MPa.^90^

However, silica aerogel has minimal mechanical characteristics and is quite fragile. Aerogel's microstructure is typically defined as a highly porous network with fractals ranging in length from 5 to 100 nm. Aerogels' elastic characteristics are often assessed using sound velocity measurements^91,92^ or static approaches.^92–94^

Silica aerogels are considered “fragile materials” because of their limited connection and large porosity in the network. The stress–strain relationship in so-called “fragile” materials develops into a “catastrophic” fracture under tension load.^95–99^

The major characteristics that govern mechanical properties in porous materials are porosity and density.

Silica aerogels are materials made up of ultrafine particles coupled in a 3D pearl necklace pattern and air-filled holes, which typically account for 85–99.8% of the total aerogel volume.^34^ As a result, when included into composites, they reduce their total overall density and hence the weight of the building envelope, while also increasing heat resistance due to their low thermal conductivity.

Silica aerogels' linked pore network is largely made up of mesopores, which have an average diameter of 20 to 40 nm.^34,65^ The sol–gel process can tailor the predominant range of pore sizes in the final material; for example, when acid catalysis conditions are used, micropores become significant, whereas the addition of organically modified silica precursors with basic moieties, such as amine groups, results in the formation of large macropores.^65,100,101^

Because of the combination of high porosity and tiny pore sizes in aerogels, the most often used traditional approach for measurement of pore structure and porosity, mercury intrusion porosimetry, is ineffective for silica aerogel. This approach is dependent on applying pressure to the material network, which in the case of aerogels causes significant volumetric compression and cracking, resulting in erroneous pore size and volume estimates. The nitrogen adsorption/desorption technique^34^ is the most commonly used for determining the pore size distribution of aerogels, and it functions at relative pressures less than one at 77 K. However, this approach has significant drawbacks, particularly when analysing samples based on density measurements to determine aerogel porosity and average pore size.^100,102^

In terms of density, silica aerogels are defined by two separate physical characteristics: bulk and skeletal density. Bulk density is the ratio of an aerogel's mass to its volume, including pores. This attribute may be achieved by weighing and measuring the dimensions of cut or shaped regular aerogel pieces. If this regularity cannot be attained, liquid or granular solids displacement can be employed to determine bulk density, as long as the filling medium does not penetrate the pores or compress the sample in the case of flexible aerogels.

When pure silica aerogels are tested, their skeletal density is often extremely near to that of amorphous silica.^103^ Lower values are predicted for organically modified silica aerogels,^104^ suggesting that the skeletal density is substantially determined by the aerogel synthesis precursors and circumstances.^105^ Heat treatment of the aerogel can also alter this feature,^106^ since it causes dihydroxylation and the elimination of network flaws. The bone density may be determined using helium pycnometry,^103^ with the material first milled to a fine powder form to reduce the number of closed pores.

Based on the foregoing, it is clear that pure silica aerogels cannot be employed as insulators due to their fragility. To alleviate this challenge, other materials can be used with aerogels to boost their mechanical strength.

### 3.3. Acoustic absorption

Sound insulation, also known as sound absorption, is one of the most essential properties of building materials used to ensure living comfort by lowering noise and noises reflected by surfaces.

Acoustic absorbers have been widely employed in noise management to reduce sound reflection from surfaces^107^ and to contrast sound transmission. According to ISO 717-1, acoustic insulation is defined by the weighted sound reduction index, which defines a building structure's capacity to prevent sound from flowing through itself and is represented in decibels.^108^

To allow for air circulation, sound absorption materials are often porous or membrane-based. To absorb sound, a material should have high porosity or holes that allow sound waves to enter the matrix and dissipate inside it owing to reverberation, frictional, and thermal losses.^108^ The holes that are completely separated from their adjacent pores are known as “closed” pores, and the design of these tiny pores may have an effect on bulk density, mechanical and thermal conductivity. Furthermore, open pores are more effective in absorbing sound energy than closed pores because they have a continuous linking channel with the external surface.^109,110^

According to ISO 10534,^111^ a laboratory scale impedance tube can be used to analyse acoustic insulation for small size samples. The sound absorption coefficient may be used to calculate the acoustic energy of materials, which was determined using Kundt's tube. Maximising the material's thickness can result in significant sound absorption coefficients at low frequencies.^112^ Acoustic performance decreases as the frequency range^113^ is measured in the octave, second, or third octave bands.^114^

The porous structure of silica aerogels influences their acoustic characteristics, which are determined by the synthesis circumstances and chemicals used.^115,116^ Caponi *et al.*^117^ concluded that when pore diameters are less than 8 nm, the major contribution to absorption comes from attenuation caused by dynamic mechanisms such as relaxation processes and two-level systems. When pore diameters exceed 8 nm, a greater sound attenuation is recorded, and this is attributed to phonon scattering by the sample's structural disorder, which is a static process. Because silica aerogels typically have pore sizes ranging from 2 to 50 nm, static and dynamic attenuation processes can coexist in these samples.

Moretti *et al.*^118^ investigated the impact of granule size and density on thermal and acoustic performance parameters of Cabot Corporation's silica granular aerogels. The results revealed that tiny granules with diameters ranging from 0.01 to 1.2 mm, which had greater density values, achieved the highest thermal and acoustic performance. At ambient temperatures, the thermal conductivity of these tiny granules was roughly 20 mW m^−1^ K^−1^, and the transmission loss (TL) at normal incidence was 19 dB at about 6400 Hz for 40 mm thickness.

Li *et al.*^119^ also investigated thermal and acoustic insulation qualities. The authors created MTES-based silica aerogels with thermal conductivities of 21.5 to 25.5 mW m^−1^ K^−1^. The silica aerogel, with a thickness of 11.8 mm and density of 60 kg m^−3^, had a sound absorption coefficient of 0.91 for sound waves at 2000 Hz frequency and a sound TL of 13–21 dB between 500 and 1600 Hz.

## 4. Classification and application of silica aerogel-based composites

Composite materials are made up of two or more chemically and physically diverse phases separated by a discrete interface. The various systems are carefully merged to produce a system with more valuable structural or functional features than any of the constituents could accomplish on its own.^120^

A composite material consists of three phases: (i) the matrix phase, also known as the continuous phase; (ii) the filler or reinforcement phase, which is surrounded by matrix material; and (iii) the interface phase, which has distinct structures and characteristics than the matrix and filler phases.

Silica aerogel-based composites are classed according on the kind of filler employed in their composition. Scientists are now employing a wide range of materials to strengthen the fragile structure of silica aerogels or to insert aerogels as a filler into the matrix of composites. Thus, the most often utilised aerogel composites are aerogel–fibre composites,^121–133^ aerogel–cement composites,^134–141^ and aerogel–polymer composites.^142–146^

In this chapter, we discuss the most researched and widely used aerogel-based composites in the construction industry, as well as current commercial goods used today in the construction and building sectors.

### 4.1. Aerogel–fibre composites

Fibre reinforcing,^147,148^ polymer cross-linking,^149^ and flexible group substitution^150^ are common ways for improving the mechanical characteristics of silica aerogels. The fibre reinforcing approach, for example, seeks to improve the mechanical characteristics of composite aerogels by using the skeletal support of the fibres as well as the fibres' impeding influence on the fracture expansion. Fibre insertion can serve as both a reinforcement for the skeletons of silica aerogels and a means of uniformizing the size of aerogel particles.

Glass fibres,^151^ silicon carbide fibres,^152^ carbon fibres,^153^ and silica nanowires^154^ are among the most prevalent inorganic fibres today. Silica nanowires, for example, are one-dimensional nanomaterials with high aspect ratios and diameters ranging from a few nanometers to a few of micrometres. Silica nanowires tend to mix into a variety of complicated morphologies as they grow. So far, silica nanowires have been effectively formed into the forms of flowers, carrots, clusters, and spheres.^155^ Silica nanowires have become a popular inorganic dopant material because to its wide source, excellent thermal stability, low coefficient of thermal expansion, strong mechanical strength, and superior chemical stability.^156^

Wu *et al.* (2021),^121^ Yuan *et al.* (2012),^122^ and Zhou *et al.* (2018)^123^ created aerogel composites with high bending (0.6–1.3 MPa) and compression strength (0.4–2.2 MPa) by combining glass fibre with silica aerogel, and a low heat conductivity of 0.024–0.026 W m^−1^ K^−1^. Although the material's strength has been significantly enhanced *via* composite material, thermal conductivity efficiency has been compromised, owing primarily to the high content of fibre and other additives in the composite materials prepared by the existing process, and achieving a high proportion of silica aerogel composite is challenging. As a result, it is difficult to manufacture fibre composite silica aerogel material with outstanding mechanical and thermal insulation qualities.

In the last several decades, the preparation process of fibre composite aerogel felt has been divided into two categories: *in situ* forming composite and secondary pressing composite. *In situ* forming composite refers to the sol–gel technique of creating silica aerogel in which the premade fibre felt or chopped fibre is immediately submerged in the sol, dried, and formed.^124^ Secondary compression moulding involves preparing silica aerogel first, followed by the application of additives and external thermal compression moulding.^125^ Yu *et al.* (2019)^126^ created a quartz fibre/Al_2_O_3_–SiO_2_ aerogel (QF/ASA) composite using the sol–gel impregnation process; the composite possesses a thermal conductivity of 0.049 W m^−1^ K^−1^ and a compressive strength of 0.85 MPa. Huang *et al.* (2018)^127^ produced an aerogel composite fibre felt by impregnating water glass and glass fibre. The composite's elastic modulus is 0.97 MPa, and its thermal conductivity is 0.0236 W m^−1^ K^−1^. Yuan *et al.* (2012)^122^ crushed silica aerogel powder and distributed glass fibre into form, and the heat transfer coefficient of composites containing 20 wt% glass fibres at 300 °C and 600 °C was 0.025 W m^−1^ K^−1^ and 0.030 W m^−1^ K^−1^, respectively.

Selver *et al.* (2021)^128^ combined silica aerogel with epoxy resin of various volume fractions, infused it into glass fabric, and vacuum dried it to form aerogel composite sheets, with a heat conductivity of 0.438 W m^−1^ K^−1^. *In situ* forming composite materials are easier to produce than secondary compression moulding, and the resulting composite materials have greater thermal insulation qualities.

Material scientists are likewise interested in the qualities of aerogel composites, and the majority of them are currently investigating the appropriate proportions of fibres/aerogels in composites to achieve greater insulating performance and mechanical stability.

**Fig. 1:**
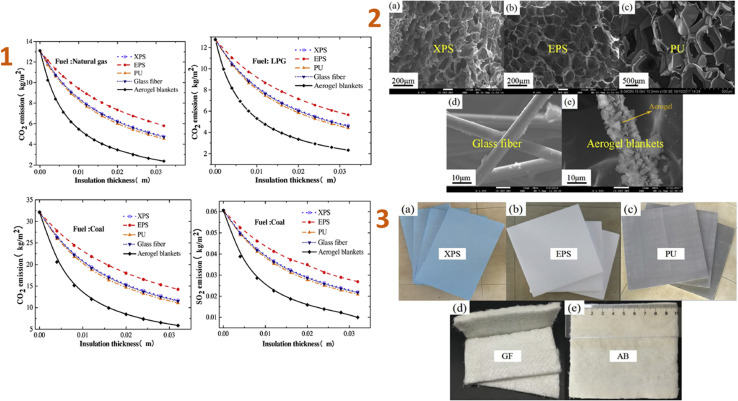
(1) Effect *versus* the thickness on CO_2_ and SO_2_ emissions of five insulation materials; (2) SEM images of five types of insulation materials: (a) XPS, (b) EPS, (c) PU foam, (d) GF, (e) aerogel blankets; (3) optical image of five types of insulation materials: (a) XPS, (b) EPS, (c) PU foam, (d) GF, (e) aerogel blankets. This figure has been adapted/reproduced from ref. 129 with permission from Elsevier under the license number 5823621056830, copyright 2024.

Huang *et al.* (2020)^129^ developed an ordinary construction model in a subtropical and humid climatic zone in China, analysed the impacts of the novel aerogel super-insulation materials, and compared them to four others regularly used insulation materials. The results indicated that aerogel had the lowest ideal insulating thickness of 3.7 mm, compared to XPS, EPS, PU, and GF, which have thicknesses of 44 mm, 70 mm, 38 mm, and 45 mm, respectively (Fig. 1).

When compared to the four most widely used building insulation materials, aerogel reduces greenhouse gas emissions quicker as thickness increases. The novel aerogel material has the potential to reduce carbon emissions by 8.169 kg per m^2^ per year, making it more environmentally friendly.

Guo *et al.* (2020)^130^ examined both moisture and heat transmission in silica aerogel/fiberglass composites and concluded that the thermal conductivity of an aerogel blanket rises linearly by 24% when the mean surface temperature fluctuates from 280 K to 300 K under dry circumstances. Moisture infiltration can dislodge aerogel from fibres, accumulating powdery particles into aggregates that are easily separated from the fibres. As a result, this occurrence can cause irreparable damage to the aerogel blanket.

**Fig. 2:**
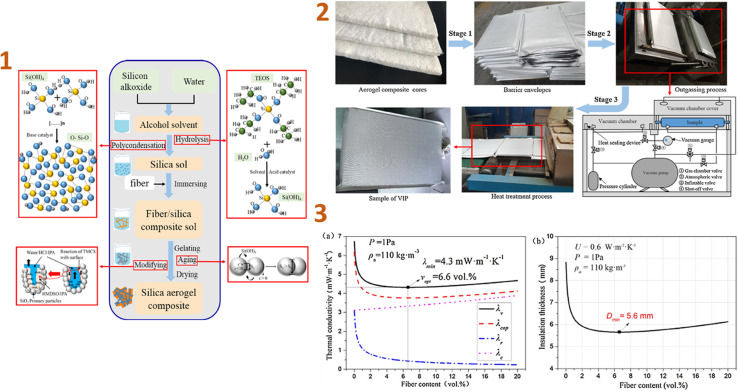
(1) Preparation schematic of aerogel composite cores; (2) preparation schematic of VIPs with aerogel composite cores; (3) thermal performance of VIPs for various fibre contents: (a) thermal conductivity, (b) insulation thickness. This figure has been adapted/reproduced from ref. 131 with permission from Elsevier under the license number 5823631121448, copyright 2024.

In the study of Wu *et al.* (2017)^131^ the thermal performance of vacuum insulation panels (VIPs) made of silica aerogel/glass fibre composites was investigated and they have concluded that the density and content of aerogels and fibres in the VIPs have a significant impact on blanket performance and service life. The coefficient of thermal insulation performance (*U*-value) was 0.6 W m^−2^ K^−1^ with a thickness of 5.6 mm when the fibre content was 6.6 vol% and the aerogel density was 110 kg m^−3^ (Fig. 2).

VIPs with aerogel composite cores often have a longer service life when the aerogel density and fibre content are lower. As a result, samples containing 1.8–2.0 vol% fibres and aerogels with densities ranging from 50 to 143 kg m^−3^ have a service life of over 50 years, which can considerably enhance the usage of VIPs in construction applications.

**Fig. 3:**
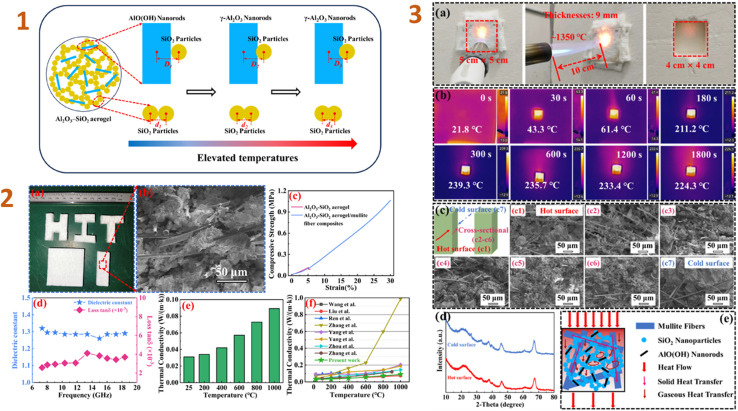
(1) Evolution schematically of the micro-morphology of Al_2_O_3_–SiO_2_ aerogel nanoparticles during high-temperature calcination; (2) machinability properties (a), SEM image (b), compressive properties (c), dielectric properties (d), and thermal conductivity (e) of Al_2_O_3_–SiO_2_ aerogel/mullite fibre composites, comparison of thermal conductivity for present work and previous reports similar composites; (3) details of the butane torch as an exposed heat source for back-temperature testing (a), infrared thermograms of back-temperature tests of composites at different times (b), SEM images of composites after back-temperature testing (c), XRD patterns of the hot and cold surfaces of the composites after back-temperature testing (d), and thermal transfer mechanism in composites (e). This figure has been adapted/reproduced from ref. 132 with permission from Elsevier under the license number 5823640531017, copyright 2024.

Zhang *et al.* (2024)^132^ studied the Al_2_O_3_–SiO_2_ aerogel composites as high-temperature thermal insulators. They employed boehmite nanorods (20–30 nm in length and 3–5 nm in diameter) as filler. Their study yielded composites with low density (260 kg m^−3^), low dielectric constant (1.28), and low dielectric loss ((2.5–4.1) × 10^−3^) (Fig. 3). To evaluate the high temperature insulation capability, a 1350 °C butane torch was employed. The composite specimens have outstanding insulation capabilities, are lightweight, and have a high thickness, making them suitable for high temperature thermal insulation-wave transparent integrated materials for hypersonic missile radomes. Tang *et al.* (2024)^157^ have also employed nanoscale fillers, such as silica nanowires. As a consequence of the research, the composites produced have good hydrophobic characteristics and high temperature tolerance. A composite has a compressive strength of 1.379 MPa at a 60% strain rate.

**Fig. 4:**
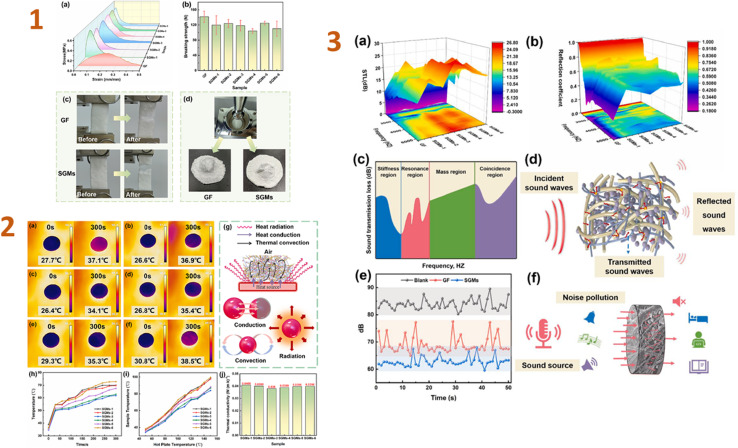
(1) (a) Tensile stress–strain curves, (b) bursting strength curves, pictures before and after (c) tension and (d) breaking of GF (glass fibre felt) and SGMs (the silica aerogel/glass fibre/hot melt fibre composites); (2) (a–f) infrared images of surface temperatures of SGMs placed on a hot plate at 50 °C; (g) heat transfer principle; (h) surface temperature profiles with time when placed on a hot plate at 100 °C; (i) surface temperature profiles for 300 s on a hot plate at different temperatures; (j) thermal conductivity of SGMs; (3) acoustic performance testing of GF and SGMs. (a) STL; (b) reflection coefficient; (c) the STL curve controlled by various regions; (d) schematic diagram of acoustic propagation; (e) sound insulation decibel curve of GF and SGMs; (f) schematic diagram of sound insulation of SGMs. This figure has been adapted/reproduced from ref. 133 with permission from Elsevier under the license number 5823641069042, copyright 2024.

In the recent study, Yang *et al.* (2024)^133^ developed silica aerogel composites with wet-laid glass fibre felt as a scaffold and examined the thermal and acoustic characteristics of the resulting materials. As a consequence, the tensile strength of a composite was 0.33 MPa, and the thermal conductivity of samples was 0.038 W m^−1^ K^−1^. The obtained composites contain a unique nano-3D network structure, which greatly increased sound insulation; for example, the sound transmission loss was 10 dB at frequencies greater than 2000 Hz (Fig. 4). They revealed that these composites may greatly reduce thermal and acoustic losses in buildings.

Furthermore, it is important to note that commercial grades of silica aerogel blankets are now available (Table 1).

**Table 1:** Existing commercial silica aerogel blankets

Product	Company
Cryogel® Z	Aspen Aerogel
ThermalWrap™	Cabot Corp.
Spaceloft®	Aspen Aerogel
Aerogel fiber board	Aspen Aerogel
Aerogel gypsum boards – types A & B	Aderma Locatelli Co.

Based on the foregoing, we can conclude that silica aerogel/fibre composites shown good mechanical and thermal conductivity qualities and have found use in the construction sector.

### 4.2. Aerogel–cement composites

To increase the thermal insulating performance of a material or composite, thermal insulators are embedded into the material matrix to prevent heat transfer through it. Because of the high thermal insulation of air (about 0.02 W m^−1^ K^−1^), air entrainment into cement-based matrix is commonly used to manufacture lightweight cement-based materials. The embedded air void volume can be up to 85% of the total material volume using either a chemical, physical, or mechanical approach, such as aerated or foamed concrete.^158,159^ Highly foamed concretes, on the other hand, have minimal mechanical characteristics, making them commonly utilised as ornamental renders or panels subjected to nil or restricted exterior stresses. In this scenario, scientists use several fillers to reduce the thermal conductivity coefficient, such as glass beads,^160^ expanded perlite,^161^ pumice aggregate,^162^ cenospheres,^163^ and aerogels.^164^

Aerogels are commonly utilised as aggregates and fine fillers in cementitious composite materials such as lightweight cement, foamed concrete, and mortar. Aerogel concrete with good mechanical characteristics can be utilised as a structural element, whereas mortar and foamed concrete can be used as a thermal insulating layer, plaster, block, or panel. Aerogel is often utilised as a volumetric replacement of fine aggregates, as well as a partial replacement of fine/coarse aggregates ranging from 0% to 60% by volume in cementitious composites.^165^

Zeng *et al.* (2018)^134^ studied lightweight cement-based composites including glass beads and nanosilica aerogels. As a consequence of their research, they determined that using nano-silica aerogels in the composition of cementitious matrix greatly reduced heat conductivity of composite. However, the compressive and flexural strengths drop as the aerogel concentration increases.

**Fig. 5:**
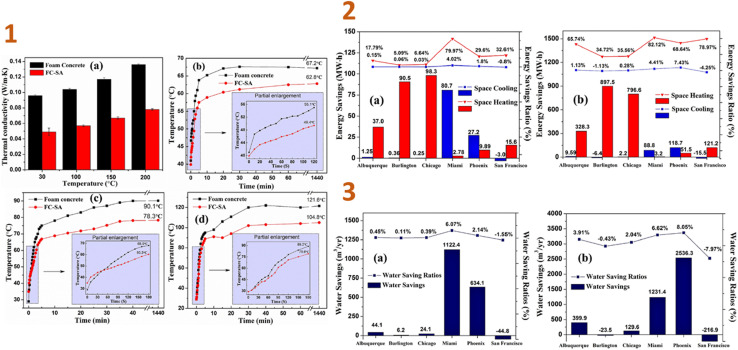
(1) Thermal conductivity test results (a) and thermal insulation performance test results ((b) 200 °C; (c) 300 °C; (d) 400 °C) of foam concrete and FC-SA; (2) space heating/cooling annual savings and saving ratios in different locations ((a) FC-SA to baseline; (b) FC-SA to B w/o insul.); (3) water savings and saving ratios from cooling tower in different locations ((a) FC-SA to baseline; (b) FC-SA to B w/o insul.). This figure has been adapted/reproduced from ref. 135 with permission from Elsevier under the license number 5823650507923, copyright 2024.

In another work of Liu *et al.* (2018),^135^ a foamed composite of concrete reinforced with silica aerogel (FC-SA) was synthesised using a vacuum impregnation approach and dried using the quick supercritical extraction method. They discovered that, based on EnergyPlus modelling findings, the FC-SA has a good energy saving impact for building envelope applications. In the wintertime, the FC-SA conserves considerable amounts of space heating energy, resulting in 90.5 MW h (5.09%) and 98.3 MW h (6.64%) energy savings in a whole winter, whereas in regions with high temperatures, this composite can not only reduce space cooling energy consumption of 80.7 MW h (6.07%) and 27.2 MW h (2.14%), but also conserve cooling water usage of 1122.4 m^3^ (6.62%) and 634.1 m^3^ (8.05%), respectively (Fig. 5).

Hanif *et al.* (2016)^136^ developed ultralightweight cement-based composites with fly ash cenospheres (FAC) and aerogel as aggregates. The addition of FAC, which also has some reactivity, improves the mechanical characteristics of the resultant composites, while the hollow structure of FAC particles and the open-porous nature of aerogel particles improves thermal insulation. Furthermore, the superior thermal insulation capabilities of aerogel-incorporated composites make them appealing for usage in buildings and construction for energy saving, but their appropriate mechanical strength (18.64–23.54 MPa) makes them appropriate for structural component applications.

Gomes *et al.* (2018)^137^ created two thermal insulating mortars: one with expanded polystyrene granules (EPS) and another with EPS mixed with silica aerogels. Thermal conductivity was found to be greater in EPS mortar than in EPS + aerogel mortar. It indicates that aerogel filler in cement mortar significantly reduces thermal conductivity coefficient in composites, transforming them into new superinsulating materials.

**Fig. 6:**
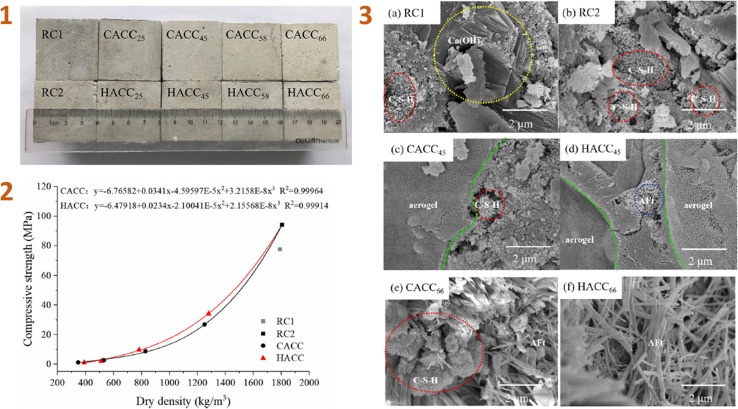
(1) Physical appearance of hardened ACCs; (2) compressive strength of ACCs; (3) SEM images of ACCs. This figure has been adapted/reproduced from ref. 138 with permission from Elsevier under the license number 5823660784831, copyright 2024.

Lu *et al.* (2020)^138^ developed aerogel/cement composites (ACCs) and classified them into two types: commercial aerogel/cement composite (CACC) and hybrid aerogel/cement composite (HACC) (Fig. 6). The primary distinction between these types of composites is the use of KH-550 silane coupling agent in the HACC.

According to the study's findings, surface modification using silane coupling agent enhanced the hydrophobicity of the composite while decreasing its specific surface area. When compared to CACC, HACC has lower thermal conductivity and higher compressive strength, indicating that this composite material has great potential in the application of thermal insulation systems in buildings, such as exterior insulation wall infrastructure, self-insulating wall systems, and roof insulation wall systems.

Bostanci *et al.* (2020)^139^ created alkali-activated slag (AAS) mortars (geopolymer) using silica aerogel powder and recycled rubber. The combination of silica powder and waste rubber resulted in reduced compressive and flexural strengths. When compared to pure aerogel loading, the loss in strength was mostly attributable to scrap rubber loading. However, increasing the quantity of aerogel in the composite increased the heat conductivity of the specimens.

**Fig. 7:**
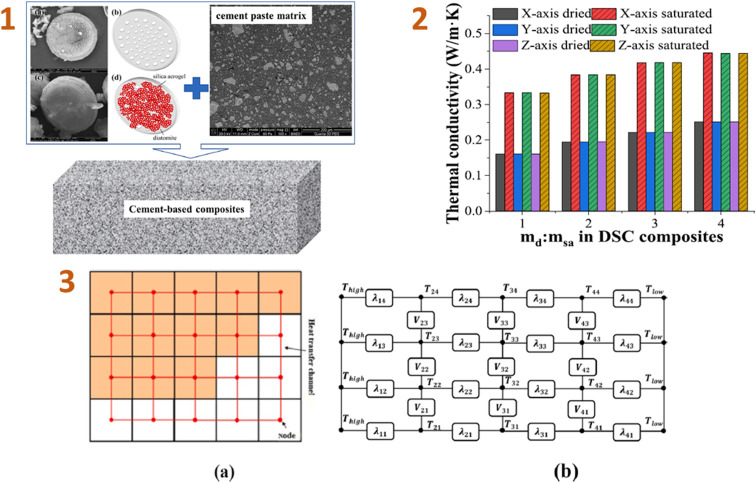
(1) Schematic diagrams and images of the cement-based composites: (a) diatomite scanning electron microscopy (SEM) photograph, (b) diatomite schematic diagram, (c) DSAC composite SEM photograph, and (d) DSAC composite schematic diagram; (2) simple two-dimensional heat transfer network schematic diagram of 4 × 5 voxels; (3) relationship between the thermal conductivity of cement-based DSAC materials and the diatomite/silica aerogel ratio in the DSAC. This figure has been adapted/reproduced from ref. 140 with permission from Elsevier under the license number 5823670120852, copyright 2024.

Liu *et al.* (2023)^140^ conducted computational simulations of the effective thermal conductivity of cement-based composites made from diatomite and silica aerogel. As a consequence, the comparison test results reveal that, while the thermal conductivity of DSAC composites differs significantly from that of the cement paste matrix, the results obtained by combining the suggested heat conduction technique with the FDM are still very consistent (Fig. 7). Increasing the diatomite/silica aerogel mass ratio of the DSAC composite reduces the total heat conductivity of cement-based DSAC materials.

**Fig. 8:**
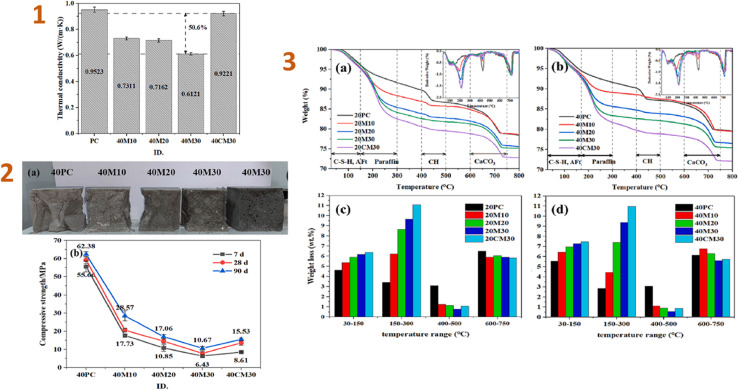
(1) Thermal conductivities of HSC (cured at 40 °C for 7 d); (2) (a) cross-sections of five samples and (b) samples cured at 40 °C for 7, 21, and 28 d; (3) (a and b) TGA curves and (c and d) weight-loss bar graphs for the PC and HSC pastes cured at 20 and 40 °C for 7 d. This figure has been adapted/reproduced from ref. 141 with permission from Elsevier under the license number 5823670811494, copyright 2024.

Shen *et al.* (2023)^141^ produced a phase-changing material based on a paraffin/silica composite and applied it to cement. The results indicated that thermal conductivity decreased when the phase-changing/silica aerogel composite ratio rose. The outcomes of several analyses revealed that the composite has a suitable phase-change temperature range (20.8–34.4 °C) and high latent heat storage (94.45 J g^−1^). The composite also demonstrated resistance to thermal deterioration below 110 °C, resulting in strong thermal stability across a normal operating temperature range. The compressive strength of the resulting composite was around 8.6 MPa (Fig. 8).

As it can be concluded, due to low thermal conductivity and superior mechanical strength, silica aerogel/cement mortars can be employed as an insulating material in the construction and building industries.

### 4.3. Aerogel–polymer composites

Because of the adaptability of silica aerogels, new composite materials are being produced by putting them into various matrices (polymer matrices) to replace more traditional materials in construction applications.

**Fig. 9:**
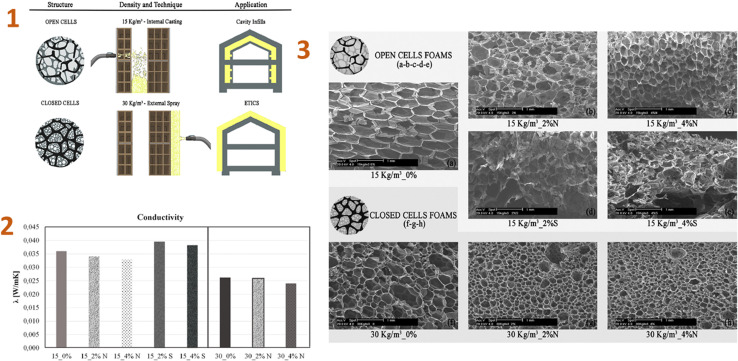
(1) Density and application technique of the foams; (2) thermal conductivity test values; (3) SEM micrographs: (a) 15 kg per m^3^ neat, (b) 15 kg per m^3^ 2% nanoclay, (c) 15 kg per m^3^ 4% nanoclay, (d) 15 kg per m^3^ 2% aerogel, (e) 15 kg per m^3^ 4% aerogel, (f) 30 kg per m^3^ neat, (g) 30 kg per m^3^ 2% nanoclay, (h) 30 kg per m^3^ 4% nanoclay. This figure has been adapted/reproduced from ref. 142 with permission from Elsevier under the license number 5823671317545, copyright 2024.

Stazi *et al.* (2019)^142^ produced the nano-foam using polyurethane (PU), silica aerogel, and montmorillonite nanoparticles. As a result, low-density foams containing 4% nanoparticles improved every characteristic, including the smallest cell diameter structure, morphological regularity, low thermal conductance, humidity sorption, and vapour permeability, as well as significantly increasing compression and tensile stiffness and strength (Fig. 9).

Patil *et al.* (2020)^143^ investigated the heat transfer properties of epoxy-based composites with two distinct fillers: aluminium hydroxide and silica aerogel. In this work, multiple models such as Maxwell's model, L–N models, Russell's model, and Agari and Uno predict reasonably well the thermal conductivity of the polymer composite. The thermal conductivity coefficients of epoxy/aluminium hydroxide composite were 0.2453–0.4894 W m^−1^ K^−1^, whereas epoxy/silica aerogel composite had coefficients ranging from 0.2453 to 0.1721 W m^−1^ K^−1^. Silica aerogel composites also displayed strong interfacial interaction between the aerogel and the polymer matrix, as well as an effective strengthening effect.

**Fig. 10:**
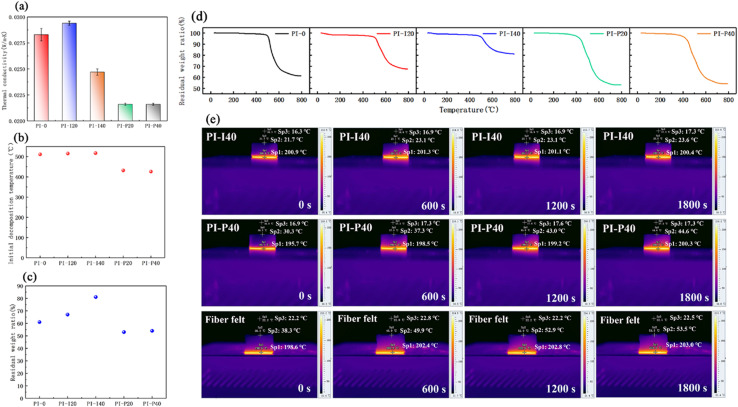
(a) Thermal conductivities, (b) Ti, (c) residual weight ratio and (d) TGA curves of the PI–silica aerogel composite aerogels. (e) Pseudo-colour thermal images of PI-I40, PI-P40 and commercial glass fibre felt on the hot plate at 200 °C. This figure has been adapted/reproduced from ref. 144 with permission from Elsevier under the license number 5823680328471, copyright 2024.

Xi *et al.* (2023)^144^ developed fire-resistant polyimide–silica aerogel composites for aerospace use. The resulting composite has low shrinkage (3.7%), low density (610 kg m^−3^), low thermal conductivity (0.0216 W m^−1^ K^−1^), and high hydrophobicity (1460). The morphology and characteristics of polyimide (PI)–silica aerogel composite aerogels are influenced by the kind and concentration of silica aerogel powder (Fig. 10).

The samples exhibit great compressibility, with no breakage occurring up to 80% strain. As the silica aerogel powder quantity rises, so does the stress at 80% strain. The incorporation of silica aerogels improves the aerogel's thermal performance, fire resistance, and structural integrity under flame, but has a negative impact on moisture resistance. Given the actual application of PI–silica aerogel composites in aerospace, the proven standard requirements of the vacuum outgassing test exhibited no evident deterioration following thermal cycling, but were marginally influenced by proton and UV irradiation. This indicates that these composites have the potential to be useful in the aerospace industry.

**Fig. 11:**
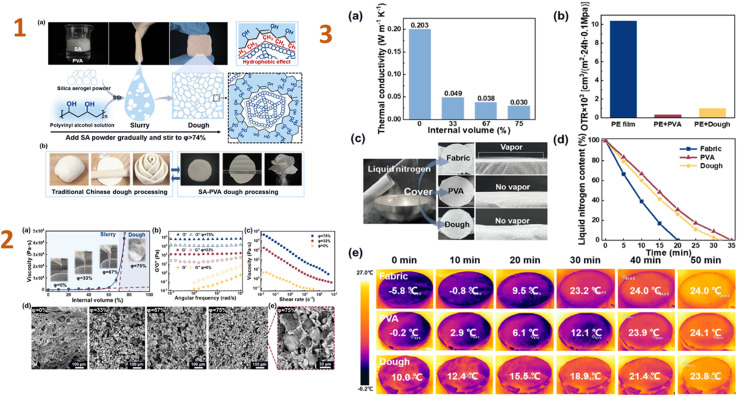
(1) Formation of the SA–PVA dough. (a) Schematic diagram of the formation of high internal phase structure in the SA–PVA dough. (b) Similar moulding properties of the SA–PVA dough to traditional Chinese dough; (2) (a) changes in viscosity during the formation of the dough. (b) Changes in *G*′ (storage modulus) and *G*′′ (loss modulus), (c) variation of viscosity with shear rate and (d and e) SEM images of the composites with different SA contents; (3) (a) thermal conductivity of as-prepared PVA/SA composite materials with different SA content. (b) A comparison of oxygen permeation rates for PE film, PVA coating, and dough coating. (c) Experimental design for thermal insulation and gas barrier properties of the dough. (d) Changes in liquid nitrogen weight over time under the encapsulation and (e) surface temperature changes over time using FLIR testing under the encapsulation of PE film, PVA coating, and dough coating. This figure has been adapted/reproduced from ref. 146 with permission from Elsevier under the license number 5823680861420, copyright 2024.

Wang *et al.* (2024)^145^ created waterproof PVA–montmorillonite/silica aerogel composites for heat insulation. As a consequence, the composite has great hydrophobic characteristics and an extremely low thermal conductivity (0.034 W m^−1^ K^−1^). The nanoporous silica network inhibits gas phase conduction, resulting in a drop in thermal conductivity. Furthermore, filled silica networks in lamellas act as stress bridges, increasing stress transmission and load-bearing capacity. In an analogous work, Zhang *et al.* (2024)^146^ studied a PVA–silica aerogel composite. In this study, they produced a PVA dough comprising 74% hydrophobic silica aerogel particles. The dough is extremely pliable, allowing for simple moulding into various shapes and displaying exceptional plasticity in later processing. Furthermore, the nanoporous structure of silica aerogel, together with the building of the PVA network, provides the dough with superior thermal insulation and oxygen barrier properties (Fig. 11).

## 5. Conclusion

Aerogels have emerged as one of the most potential materials for thermal insulation in the past few decades, therefore, they are increasingly being used to replace traditional materials in a variety of matrix. Thus, this paper addressed current achievements in incorporating silica aerogels into composites and building constructions. As demonstrated, aerogel-containing materials are already being widely produced and tested for construction applications such as panels, blankets, mortars, cement, plasters, and so on.

When silica aerogels are added to the newly created materials, the insulating qualities improve significantly. The thermal conductivities vary between 0.014 and 0.026 W m^−1^ K^−1^ when the aerogel is applied in the form of vacuum insulation panels or blankets, and can be up to one order of magnitude higher when the aerogel is incorporated in cement, mortars or concrete, with the increase obviously dependent on the amount of aerogel in the mixture. In general, the addition of aerogels to various construction materials results in significant decreases in composite densities and thermal conductivities when compared to systems without aerogels.

However, this inclusion often has a detrimental impact on mechanical qualities. Because the compressive strengths of composites decrease as the content of silica aerogels increases, the search for the optimal quantity of aerogel is required in this circumstance. To maximise the mechanical stability of composites, additional filler may be required in addition to silica aerogels.

Furthermore, because aerogels improve energy conservation in the building's envelope, they result in a considerable decrease in greenhouse gas emissions when compared to typical building insulation materials such as XPS, EPS, and PU.

Given the foregoing, we may infer that silica aerogel-based composites have a high potential for utilisation in the construction and building industries due to their unique features and availability.

## Data availability

The authors confirm that the data supporting the findings of this study are available within the article.

## Conflicts of interest

There are no conflicts to declare.
